# Encapsulated Mulberry Fruit Extract Alleviates Changes in an Animal Model of Menopause with Metabolic Syndrome

**DOI:** 10.1155/2019/5360560

**Published:** 2019-04-28

**Authors:** Jintanaporn Wattanathorn, Supannika Kawvised, Wipawee Thukham-mee

**Affiliations:** ^1^Department of Physiology, Faculty of Medicine, Khon Kaen University, 40002, Thailand; ^2^Integrative Complementary and Alternative Medicine Research and Development Center, Khon Kaen University, 40002, Thailand; ^3^Research Institute for Human High Performance and Health Promotion, Khon Kaen University, 40002, Thailand

## Abstract

Currently, the therapeutic strategy against metabolic syndrome and its complications is required due to the increasing prevalence and its impact. Due to the benefits of both mulberry fruit extract and encapsulation technology, we hypothesized that encapsulated mulberry fruit extract (MME) could improve metabolic parameters and its complication risk in postmenopausal metabolic syndrome. To test this hypothesis, female Wistar rats were induced experimental menopause with metabolic syndrome by bilateral ovariectomy (OVX) and high-carbohydrate high-fat (HCHF) diet. Then, they were orally given MME at doses of 10, 50, and 250 mg/kg BW for 8 weeks and the parameters, such as percentage of body weight gain, total cholesterol, triglycerides, HDL-C, LDL-C, atherogenic index, fasting blood glucose, plasma glucose area under the curve, serum angiotensin-converting enzyme (ACE), oxidative stress status, histology, and protein expression of PPAR-*γ*, TNF-*α*, and NF-*κ*B in adipose tissues were determined. MME improved body weight gain, adiposity index, glucose intolerance, lipid profiles, atherogenic index, ACE, oxidative stress status, and protein expression of TNF-*α* and NF-*κ*B. Moreover, MME attenuated adipocyte hypertrophy and enhanced PPAR-*γ* expression. Taken altogether, MME decreased metabolic syndrome and its complication via the increased PPAR-*γ* expression. Therefore, MME is the potential candidate for improving metabolic syndrome and its related complications. However, further research in clinical trial is still necessary.

## 1. Introduction

Metabolic syndrome (MetS), one of the important noncommunicating diseases (NCDs), is continually increasing. It has been reported that the global prevalence of MetS in female is higher than that in male. It has been reported that the prevalence of MetS in male and female are around 23% and 29%, respectively [1]. This prevalence is also elevated in postmenopausal women [2, 3]. In addition, postmenopausal condition increases vulnerability to many metabolic disorders, including obesity, hypertension, insulin resistance, glucose intolerance, and dyslipidemia [4]. Moreover, it has been reported that postmenopausal metabolic syndrome is also associated with the development of adipose tissue oxidative stress and inflammation [5]. Even though the increasing rate of metabolic syndrome is alarming its importance to the world, the current therapeutic efficacy is still limited [6–8]. Therefore, a novel protective strategy against MetS that is cheap and easy to approach is required.

Recently, it has been demonstrated that substances that are rich in polyphenolic compounds, especially anthocyanins, can improve metabolic disorders in menopause [9–11]. Therefore, the application of anthocyanin-rich substances against menopause-related disorders, such as MetS and fatty liver, has gained much attention. Ripened mulberry fruits (*Morus alba* L.) are rich in anthocyanins and possess antioxidant [12–14], antidyslipidemia [15–17], antidiabetes [12, 16–18], antiobesity [19], anti-inflammation [19], and antiartherosclerosis [20] effects. In addition, several studies have demonstrated that mulberry extract can also attenuate oxidative stress-related disorders [21, 22].

Despite numerous health benefits, active ingredients in mulberry fruit, such as polyphenolic compounds including anthocyanins and flavonol glycosides, are unstable and highly labile [23, 24]. In addition, most of these substances are poorly absorbed and are instable during food processing, distribution, or storage or in the gastrointestinal tract [25–27]. All of these limitations could be solved by the formulation process. Interestingly, encapsulation technology which protects the core material by using a carrier wall can increase the stability by decreasing the decay induced by the environment, increase the solubility, and mask the undesirable taste [27, 28]. Due to the advantages of polyphenol-rich substances and mulberry fruits together with the benefit of encapsulation technology on the bioavailability mentioned earlier, we hypothesized that microencapsulated mulberry fruit extract could improve metabolic syndrome in menopause. To elucidate this issue, this study is aimed at determining the effect of the microencapsulated mulberry fruit extract on metabolic disorders in ovariectomized (OVX) rats fed with high-carbohydrate high-fat diet (HCHF), an animal model of menopausal women with metabolic syndrome.

## 2. Materials and Methods

### 2.1. Preparation of Encapsulated Mulberry Fruit Extract

Ripened mulberry fruits (*Morus alba* L. var. Chiangmai) were collected from the Queen Sirikit Department of Sericulture Center, Udon Thani Province. The fresh mulberry fruits were cleaned and dried in the oven (Memmert GmbH, USA) at 60°C for 72 hours. The dried mulberry was grounded to fine powder and prepared as 50% alcohol extract by the maceration technique. The extract was filtered with Whatman No.1 filter paper and dried in the oven (Memmert GmbH, USA) at 60°C for 24 hours. In this study, maltodextrin dextrose equivalent 10 (DE10) was selected as the encapsulation matrix. It was mixed with mulberry fruit extract at the ratio of 9 : 1 (*w*/*w*). Then, the mixture was dissolved in warm distilled water at 50°C and stirred for 30 minutes. Following this process, the solution was frozen at -20°C for 18 hours in a freezer and subjected to a drying process in a freeze dryer (Labconco freeze dryer, Labconco Corporation, Kansas City, MO, USA) for 48 hours (−86°C, 0.008 mbar). The dry sample was packed and stored in a desiccator containing silica gel at 4°C.

### 2.2. Measurement of Total Phenolic Compound Contents and Flavonoid Contents

The amount of total phenolic compounds in the sample was determined by using the Folin-Ciocalteu colorimetric method via a microplate reader (iMark™ Microplate Absorbance Reader) [29]. The freshly prepared reagent consisting of 158 *μ*l of distilled water and 20 *μ*l of 50% *v*/*v* Folin-Ciocalteu reagent (Sigma-Aldrich, USA) was mixed with 20 *μ*l of the extract and incubated for 8 minutes. Following this process, 30 *μ*l of 20% sodium carbonate (Na_2_CO_3_) (Sigma-Aldrich, USA) was added and incubated at room temperature in a dark room for 2 hours. Then, absorbance was measured at 765 nm. Results were expressed as mg gallic acid equivalent (GAE)/mg extract. Various concentrations of gallic acid (Sigma-Aldrich, USA) were used as a standard calibration curve.

Flavonoid content was assessed by using the aluminum chloride method [30]. In brief, 100 *μ*l of the extract at various concentrations was mixed with 100 *μ*l of 2% methanolic aluminum chloride (Sigma-Aldrich, USA) and incubated at room temperature in a dark room for 30 minutes. At the end of the incubation period, absorbance at 415 nm was taken against the suitable blank. Various concentrations of quercetin (Sigma-Aldrich, USA) were used for the standard calibration curve preparation. Results were expressed as *μ*g quercetin equivalent/mg extract.

### 2.3. Fingerprint Chromatogram Assessment

High-performance liquid chromatography (HPLC) analysis was used to determine the fingerprint chromatogram. Chromatography was performed by using a Waters® system equipped with a Waters® 2998 photodiode array detector. Chromatographic separation was performed using a Purospher® STAR, C-18 encapped column (5 *μ*m) and LiChroCART® 250-4.6 HPLC cartridge, Sorbet Lot No. HX255346 (Merck, Germany). The mobile phase (HPLC-grade) consisted of 100% methanol (solvent A) (Fisher Scientific, USA), and 2.5% acetic acid (solvent B) (Fisher Scientific, USA) in deionized (DI) water was used to induce gradient elution. The gradient elution was carried out at a flow rate of 1.0 ml/min with the following gradient: 0-17 min, 70% A, 18-20 min, 100% A; 20.5-25 min, 10% A. The sample was filtered (0.45 *μ*m, Millipore), and a direct injection of the tested sample at the volume of 20 *μ*l on the column was performed. Chromatogram detection was performed at 280 nm using a UV detector, and data analysis was performed using EmpowerTM3.

### 2.4. Determination of Biological Activities

#### 2.4.1. Antioxidant Activity Assessment

Antioxidant activity of the extracts was determined by using 1,1-diphenyl-2-picryl-hydrazyl (DPPH), ferric reducing antioxidant power (FRAP), and 2,2′-azino-bis(3-ethylbenzothiazoline-6-sulfonic acid) (ABTS) assays. For the DPPH assay, an aliquot of 0.15 mM DPPH in methanol (180 *μ*l) (Sigma-Aldrich, USA) was mixed with 20 *μ*l of various concentrations of extracts and incubated for 30 minutes. The absorbance was measured against the blank at 517 nm via a microplate reader (iMark™ Microplate Absorbance Reader) [31].

FRAP assays were carried out based on the ability of the tested substance to convert ferric tripyridyltriazine (Fe^3+^-TPTZ) to ferrous tripyridyltriazine (Fe^2+^-TPTZ). The FRAP working solution was prepared by mixing 300 mM acetate buffer (Sigma-Aldrich, USA), 10 mM TPTZ (Sigma-Aldrich, USA), and 20 mM ferric chloride (FeCl_3_) (Sigma-Aldrich, USA) solutions at a ratio of 10 : 1 : 1, respectively. In brief, 190 *μ*l of the FRAP reagent was mixed with 10 *μ*l of extract and incubated at 37°C for 10 minutes. After the incubation, an absorbance at 593 nm was measured against the blank [32]. Ascorbic acid was used as positive control, and results were expressed as EC_50_ value.

2,2′-Azino-bis(3-ethylbenzothiazoline-6-sulfonic acid) (ABTS) was also used to determine the free radical scavenging activity of the microencapsulated mulberry fruit extract [33]. The ABTS solution was prepared by mixing 7 mM ABTS (Sigma-Aldrich, USA) and 2.45 mM potassium persulfate (K_2_S_2_O_8_) (Sigma-Aldrich, USA) at a ratio of 2 : 3 (*v*/*v*) and diluted with deionized water at a ratio of 1 : 20 (*v*/*v*) before use. In brief, 30 *μ*l of various concentrations of extracts was mixed with 120 *μ*l of distilled water and 30 *μ*l of ethanol and reacted with 3 ml of ABTS solution. The absorbance was measured at 734 nm with a spectrophotometer (Pharmacia LKB-Biochrom 4060). Trolox was used as a standard. Results were expressed in terms of EC_50_ (concentration in micrograms per milliliter required to inhibit ABTS radical formation by 50%).

#### 2.4.2. Measurement of Carbohydrate-Metabolizing Enzymes

The effects of extract on carbohydrate-metabolizing enzymes were assessed by focusing on the alterations of *α*-amylase, *α*-glucosidase, and aldose reductase enzymes. The assessment of *α*-amylase inhibition activity was measured using the spectrophotometric method with slight modification [34]. In brief, the substrate solution was prepared by dissolving 1 g of starch (Univar) and 0.01 M calcium chloride (CaCl_2_) (Sigma-Aldrich) in 100 ml of 0.5 M Tris-HCl buffer, pH 6.9 (Sigma-Aldrich), boiled for 5 minutes, and incubated at 37°C for 5 minutes before use. Then, the reaction mixture consisting of 25 *μ*l of porcine pancreatic amylase (2 units/ml, Sigma-Aldrich) and 50 *μ*l of substrate solution was mixed with 50 *μ*l of various concentrations of extracts and incubated at 37°C for 10 minutes. The reaction was stopped by adding 125 *μ*l of 50% acetic acid and centrifuged at 3000 rpm, 4°C, for 5 minutes. The supernatant was harvested and absorbance measured at 595 nm.

The inhibitory effect of extract on *α*-glucosidase was determined by the chromogenic method [35]. A stock solution of *α*-glucosidase was prepared by dissolving 2 units of *α*-glucosidase (Sigma-Aldrich) in 1 ml of 50 mM phosphate buffer, pH 6.8. The reaction mixture containing 20 *μ*l of various concentrations of the sample, 60 *μ*l of *α*-glucosidase enzyme, and 20 *μ*l of 1 mM para-nitrophenyl-glucopyranoside (Sigma-Aldrich) was incubated at 37°C for 20 minutes. Following this process, the reaction was stopped by adding 50 *μ*l of 1 M sodium carbonate (Sigma-Aldrich) and absorbance was measured at 405 nm.

Aldose reductase activity was measured via a spectrophotometric method by measuring the reduction in the absorption of NADPH at 390 nm over a 4-minute period with 10 mM DL-glyceraldehyde as the substrate [36]. The tissue sample was isolated and prepared as homogenate in 100 mM potassium phosphate buffer, pH 6.2, and subjected to a 10,000 rpm centrifugation at 4°C for 30 minutes. The supernatant was harvested and served as the source of aldose reductase. The assay mixture containing 100 *μ*l of the aldose reductase enzyme, 300 *μ*l of 0.1 M sodium phosphate buffer (pH 6.2), 100 *μ*l of 0.15 mM NADPH (Sigma-Aldrich), 300 *μ*l of 10 mM DL-glyceraldehyde, and 100 *μ*l of different concentrations of the extract was recorded at the absorbance of 390 nm with a spectrophotometer (Pharmacia LKB-Biochrom 4060) as T0. After 4 minutes of incubation period, the absorbance was recorded as T4. Aldose reductase activity was calculated and expressed in terms of EC_50_ (concentration in micrograms per milliliter required to inhibit enzyme activity by 50%). Based on the suppression effect of quercetin on aldose reductase in the previous study, it was used as positive control [36].

#### 2.4.3. The Suppression Effect on Angiotensin-Converting Enzyme

The assessment was performed based on the cleavage of the substrate hippuryl-glycyl-glycine by ACE and subsequent reaction with 2,4,6-trinitrobenzenesulfonic acid (TNBS) to form 2,4,6-trinitrophenyl-glycyl-glycine [37]. In this study, the angiotensin-converting enzyme from rabbit lungs (Sigma, USA) was used as enzyme source and samples were prepared at various concentrations. An aliquot of the angiotensin-converting enzyme (0.05 units/ml, 10 *μ*l) was mixed with various concentrations of the extract at the volume of 20 *μ*l and served as an experimental sample or mixed with phosphate buffer (5 mM, pH 8.3) and served as control. Captopril was used as a positive control. Following this process, 50 *μ*l of 100 mM Hip-Gly-Gly (Sigma, USA) was mixed with the reaction mixture and incubated at 37°C for 35 minutes. The reaction was stopped by adding 120 *μ*l of 3 M sodium tungstate (Sigma, USA) and 0 .5M sulfuric acid (Sigma, USA) and centrifuged at 2500 rpm for 10 minutes. After the centrifugation, an aliquot of the supernatant was placed into a 96-well microtiter plate, mixed with 20 *μ*l of 60 mM TNBS (Sigma, USA), and incubated in dark conditions for 20 minutes. At the end of the incubation period, an absorbance at 415 nm was recorded with a microplate reader (iMark™ Microplate Absorbance Reader). Results were expressed as EC_50_.

#### 2.4.4. Assessment of Pancreatic Lipase Activity

The working solution of lipase at a concentration of 10 mg/ml was prepared by dissolving lipase from porcine pancreas type II (Sigma, USA) in deionized (DI) water and subjected to a 16,000 rpm centrifugation for 5 minutes. The supernatant was harvested for further use. In this study, 100 mM Tris buffer pH 8.2 and p-nitrophenyl laurate (pNP laurate) were used as the substrate. The pNP laurate was dissolved in 5 mM sodium acetate (pH 5.0) containing 1% Triton X-100 to produce 0.08% *w*/*v* substrate solution and served as stock solution. This solution was heated in boiling water for 1 minute, mixed well, and cooled down at room temperature. The reaction mixture containing 70 *μ*l of assay buffer, 90 *μ*l of substrate solution, 30 *μ*l of lipase, and 10 *μ*l of the extracts was mixed and incubated at 37°C for 2 hours. At the end of the incubation period, the solution was centrifuged at 16,000 rpm for 1 minute and absorbance was measured with a microplate reader (iMark™ Microplate Absorbance Reader) at 400 nm [38]. In this study, orlistat was used as positive control.

#### 2.4.5. Assessment of Cyclooxygenase-2 (COX-2) Activity

COX-2 inhibition was measured by using a colorimetric COX-2 inhibitor screening assay kit (Cayman Chemical, USA). COX-2 inhibition activity was performed according to the manufacturer's protocol. In brief, COX-2 working solution was prepared by dissolving COX-2 substance in 100 mM Tris-HCl buffer, pH 8.0, at a ratio of 1 : 100. The reaction mixture containing 150 *μ*l of assay buffer, 10 *μ*l of extracts, 10 *μ*l of heme (Cayman Chemical, USA), 10 *μ*l of COX-II working solution, 20 *μ*l of 10 *μ*M TMPD (N,N,N′,N′-tetramethyl-p-phenylenediamine dihydrochloride) (Sigma, USA), and 20 *μ*l of 100 *μ*M arachidonic acid (Cayman Chemical, USA) was added to 96-well microtiter plates and incubated at room temperature for 30 minutes. At the end of the incubation period, an absorbance at 590 nm was recorded and results were expressed as EC_50_ [39]. Indomethacin was used as a reference compound.

### 2.5. Experimental Protocol

A total of 48 female Wistar rats (weighing 200-250 g, 10 weeks old) were obtained from the National Laboratory Animal Center, Salaya, Nakhon Pathom, Thailand. The rats were kept under standard laboratory conditions with food and water ad libitum and housed in standard metal cages (6 per cage). Temperature was controlled at 23 ± 2°C on a 12 : 12 h light-dark cycle. All procedures and experimental protocols were approved by the Institutional Animal Ethics Committee of Khon Kaen University (record no. AEKKU 27/2017). After 1 week of acclimatization, the animals were divided into 8 groups as the following.

Group I: Normal diet (ND) + vehicle: all rats in this group received normal diet and were treated with vehicle.
Group II: HCHF + vehicle: all rats in this group received high-carbohydrate high-fat diet (HCHF) diet and treated with vehicle.
Group III: OVX-HCHF diet + vehicle: all animals in this group were subjected to bilateral ovariectomy (OVX), received HCHF diet, and treated with vehicle.
Group IV: OVX-HCHF diet + isoflavone: rats in this group were subjected to bilateral ovariectomy, fed with HCHF diet, and treated with soy isoflavone extract (Fisiogen®) at dose of 15 mg/kg BW.
Group V: OVX-HCHF diet + L-carnitine: rats in this group were subjected to bilateral ovariectomy, fed with HCHF diet, and treated with L-carnitine at a dose of 250 mg/kg BW.
Group VI-VIII: OVX-HCHF diet + encapsulated mulberry fruit extract (MME): all rats in these groups were subjected to OVX, received HCHF diet, and treated with MME at various doses ranging from 10 to 50 to 250 mg/kg BW.

In this study, all OVX rats were anesthetized with thiopental sodium at a dose of 40 mg/kg BW prior to the induction of experimental menopause by bilateral ovariectomy. In brief, rats were placed in prone position and fixed to the operating table. Ovariectomy was performed by two dorsolateral incisions, approximately 1 cm long above the ovaries. The bulged area on the back was shaved bilaterally, and the skin and muscle were dissected. After the muscle dissection, the peritoneal space and adipose tissue surrounding the ovary were exposed. After identifying the ovary and uterine horn, ligation was performed at the distal uterine horn to remove the ovarian tissue completely in one action. The horn was returned to the abdominal cavity, and the muscle and skin were sutured [40, 41]. Then, the OVX rats were returned to their cage, after postoperation care. After 1 week of operation, animals were fed with a normal diet (a standard laboratory diet No. 082, C.P. Company, Bangkok, Thailand) which contained total energy around 4.02 kcal/g (fat 19.77%, protein 28.24%, and carbohydrate 51.99%) or high-carbohydrate high-fat (HCHF) diet which contained total energy around 4.62 kcal/g (fat 31.54%, protein 20.25%, and carbohydrate 48.21%). All animals were fed with either normal diet or HCHF diet for 20 weeks, and rats received HCHF diet which showed a percent change of body weight more than 40 percent; the homeostasis model assessment-estimated insulin resistance (HOMA-IR) index higher than that in the control group was selected for further study. Then, the recruited animals were randomly assigned to various treatment groups, including vehicle or distilled water, isoflavone, L-carnitine, and MME at various doses ranging from 10 to 50 to 250 mg/kg BW (the total volume of administration is 0.5 ml). All animals' food intake and body weight changes were monitored every week. In addition, the parameters of metabolic syndrome, including total plasma cholesterol, triglycerides, low-density lipoprotein (LDL), high-density lipoprotein (HDL), atherogenic index (AI index), and fasting plasma glucose were determined, and an oral glucose tolerance test (OGTT) was performed both at 4 weeks and at 8 weeks. At the end of the study period, all animals were sacrificed and fat pads were isolated from various areas, including subcutaneous fat and intra-abdominal fat (gonadal, mesenteric, and retroperitoneal fat). The adipose tissue was kept cool in ice buckets and stored at -80°C until used. The adiposity index and histology of adipocytes were determined. Serum biochemicals, including angiotensin-converting enzyme, were also assessed. In addition, the oxidative stress status of adipose tissue was also determined using the malondialdehyde (MDA) level and the activities of main scavenging enzymes, including superoxide dismutase (SOD), glutathione peroxidase (GSH-Px), and catalase (CAT). Moreover, the expression of PPAR-*γ*, TNF-*α*, and NF-*κ*B in adipose tissues was also determined.

### 2.6. Biochemical Assays

#### 2.6.1. Plasma Lipid Profile Assessment

Total plasma cholesterol was determined by using the “CHOD-PAP” enzymatic photometric test [42]. An aliquot of the sample or calibrator at the volume of 10 *μ*l was mixed with 1,000 *μ*l of cholesterol FS Reagent (DiaSys Diagnostic Systems GmbH, Germany) and incubated at 25°C in a dark room for 20 minutes. Following an incubation period, an absorbance at 500 nm was measured within 60 minutes by using a UV spectrophotometer (Pharmacia LKB-Biochrom 4060). Total cholesterol was expressed as mg/dl and calculated as follows:

(1) $$\begin{array}{c} \text{Cholesterol}\left[\text{mg}/\text{dL}\right]=\left(\text{A sample}/\text{A calibrator}\right)\times \text{Conc.Cal.}\left[\text{mg}/\text{dL}\right]. \end{array}$$

Plasma triglyceride level was determined with the enzymatic colorimetric test by using glycerol-3-phosphate oxidase (GPO) reagent (DiaSys Diagnostic Systems GmbH, Germany). In brief, 10 *μ*l of the plasma or calibrator and 1,000 *μ*l of reagent were mixed and incubated at 25°C in the dark room for 10 minutes. The absorbance was measured at 500 nm within 60 minutes by using a UV spectrophotometer (Pharmacia LKB-Biochrom 4060). Triglyceride level was expressed as mg/dl and calculated as follows:

(2) $$\begin{array}{c} \text{Triglycerides}\left[\text{mg}/\text{dL}\right]=\left(\text{A sample}/\text{A calibrator}\right)\times \text{Conc.Cal.}\left[\text{mg}/\text{dL}\right]. \end{array}$$

Plasma LDL-C was determined with the Friedewald equation [43] by using LDL-C select FS Reagent 1 and 2 (DiaSys Diagnostic Systems GmbH, Germany). In brief, 5 *μ*l of plasma or TruLab L calibrator and 280 *μ*l of reagent 1 were mixed and incubated at 37°C for 5 minutes. After incubation, an absorbance at 595 nm (A1) was measured by using the microplate reader (iMark™ Microplate Absorbance Reader). Then, 70 *μ*l of reagent 2 was added to the mixture and incubated at 37°C for 5 minutes. After incubation, the absorbance at 595 nm (A2) was measured. LDL-C was expressed as mg/dl and calculated as follows:

(3) $$\begin{array}{c} \text{LDL}-\text{C}\left[\text{mg}/\text{dl}\right]=\left(\text{A sample}/\text{A calibrator}\right)\times \text{Conc.Cal.}\left[\text{mg}/\text{dl}\right],\\ \text{A}=\left[\left(\text{A}2–\text{A}1\right)\text{sample or calibrator}\right]-\left[\left(\text{A}2–\text{A}1\right)\text{blank}\right]. \end{array}$$

Plasma HDL was determined by using HDL-C select FS Reagent 1 and 2 (DiaSys Diagnostic Systems GmbH, Germany) based on the same basic principle as that of cholesterol determination [44]. In brief, 5 *μ*l of plasma or TruLab L calibrator and 240 *μ*l of reagent 1 were mixed and incubated at 37°C for 5 minutes. After incubation, an absorbance at 595 nm (A1) was measured by using the microplate reader (iMark™ Microplate Absorbance Reader). Then, 60 *μ*l of reagent 2 was added to the mixture and incubated at 37°C for 5 minutes. After incubation, the absorbance at 595 nm (A2) was measured. HDL-C was expressed as mg/dl and calculated as follows:

(4) $$\begin{array}{c} \text{HDL}-\text{C}\left[\text{mg}/\text{dl}\right]=\left(\text{A sample}/\text{A calibrator}\right)\times \text{Conc.Cal.}\left[\text{mg}/\text{dl}\right],\\ \text{A}=\left(\text{A}2–\text{A}1\right)\text{ sample or calibrator}. \end{array}$$

#### 2.6.2. Determination of Atherogenic Index (AI)

The atherogenic index (AI index) is the most reliable indicator for the prediction of cardiovascular disease risk. The AI index was calculated by the total cholesterol (TC)/high-density lipoprotein cholesterol (HDL-C) ratio [45].

#### 2.6.3. Assessment of Fasting Plasma Glucose and Oral Glucose Tolerance Test (OGTT)

To determine the fasting plasma glucose level, all animals were fasted for 12 hours. After the food deprivation period, basal blood glucose concentrations were measured by collected blood samples from the tail vein using the Accu-Chek® Performa blood glucose meter.

According to the determination of the oral glucose tolerance test, the animals were administered 40% glucose solution at a dose of 2 g/kg of BW by oral gavage. The tail vein blood samples were taken at 30, 60, 90, and 120 minutes after the glucose administration [46]. The plasma glucose area under the curve (AUC) was calculated by trapezoidal approximation of plasma glucose levels and expressed as mg h/dl.

#### 2.6.4. Determination of Plasma Angiotensin-Converting Enzyme Activity

Plasma angiotensin-converting enzyme assay was performed by using the modified method of Serra et al. [37]. The enzymatic reaction was started by adding 20 *μ*l of plasma into 50 *μ*l of substrate solution Hip-Gly-Gly (100 mmol/l) (Sigma, USA) and incubated at 37°C for 35 minutes. The reaction was stopped by adding 120 *μ*l of 3 M sodium tungstate (Sigma, USA) and 0 .5M sulfuric acid (Sigma, USA) and centrifuged at 2500 rpm for 10 minutes. After the centrifugation, an aliquot of the supernatant was placed into a 96-well microtiter plate and mixed with 20 *μ*l of 60 mM TNBS (Sigma, USA), incubated in dark conditions for 20 minutes. At the end of the incubation period, an absorbance at 415 nm was recorded with the microplate reader (iMark™ Microplate Absorbance Reader). The standard calibration curve was prepared by using the angiotensin-converting enzyme (Sigma-Aldrich, USA) at the concentration range of 0.001-1 units/ml. Results were expressed as units/mg protein.

### 2.7. Measurement of Serum Estradiol

The blood sample of each animal was collected. Then, it was centrifuged immediately at 2000 × *g* at 4°C for 15 min. The obtained serum was kept at −80°C until the time of measurement use. The estradiol level in the samples was measured using an Estradiol DSL-4400 Radioimmunoassay kit (Diagnostic Systems Laboratories Inc., Webster, TX, USA) according to the manufacturer's instructions.

### 2.8. Histological Procedure and Adiposity Assessment

After the scarification, fat pads from various areas, including subcutaneous, gonadal, mesenteric, and retroperitoneal areas, were removed and immersed into a fixative solution containing 10% formalin (Sigma-Aldrich, USA) for 72 hours. Serial sections of tissues were cut frozen on a cryostat (Thermo Scientific™ HM 525 Cryostat) at 10 *μ*m thickness. All sections were picked up on slides coated with 0.3% aqueous solution of gelatin containing 0.05% aluminum potassium sulfate (Sigma-Aldrich, USA). All assessments were observed after being hematoxylin-eosin- (H&E-) (Sigma-Aldrich, USA) stained and photographed [47]. Adipocyte cell diameter and density were estimated from 3 randomly selected different fields of each area by using Olympus light microscope model BH-2 (Japan) under 40x magnification with the PixeLINK PL-A6xx Capture and IT tool program. In addition, the adiposity index was calculated by the sum of intra-abdominal fat weight/body weight ratio × 100 and expressed as adiposity percentage.

### 2.9. Assessment of Oxidative Stress Status in Adipose Tissues

Adipose tissues were isolated and homogenized with 0 .1M potassium phosphate buffer solution, pH 7.4 (sample dilution 10 mg: PBS 50 *μ*l). The derived homogenate was used for the determination of oxidative status, including malondialdehyde (MDA) level and the activities of superoxide dismutase (SOD), catalase (CAT), and glutathione peroxidase (GSH-Px). The protein concentrations in adipose tissue homogenate were determined by using a Thermo Scientific NanoDrop 2000c spectrophotometer (Thermo Fisher Scientific, Wilmington, Delaware, USA), and the optical density at the wavelength of 280 nm was measured.

MDA level homogenate was assessed by thiobarbituric acid reaction [48]. The reaction mixture containing 50 *μ*l of tissue homogenate, 50 *μ*l of 8.1% sodium dodecyl sulfate (SDS) (Sigma-Aldrich, USA), 375 *μ*l of 0.8% of thiobarbituric acid (TBA) (Sigma-Aldrich, USA), 375 *μ*l of 20% acetic acid (Sigma-Aldrich, USA), and 150 *μ*l of distilled water (DW) was boiled at 95°C in the water bath for 60 minutes. After boiling, it was cooled with tap water. Then, 250 *μ*l of DW and 1,250 *μ*l of the solution containing *n*-butanol and pyridine (Merck, Germany) at the ratio of 15 : 1 were added, mixed together, and centrifuged at 4000 rpm for 10 minutes. The upper layer was separated, and the absorbance was measured at 532 nm. TMP (1,1,3,3-tetramethoxypropane) (0-15 *μ*M) (Sigma-Aldrich, USA) was served as standard, and the level of MDA was expressed as ng/mg protein.

The determination of SOD activity was performed according to the method of Sun et al. [49]. In brief, 20 *μ*l of tissue sample was mixed with 200 *μ*l of the reaction mixture containing 57 mM phosphate buffer solution (KH_2_PO_4_) (Sigma-Aldrich, USA), 0.1 mM EDTA (Sigma-Aldrich, USA), 10 mM cytochrome C (Sigma-Aldrich, USA) solution, 50 *μ*M of xanthine (Sigma-Aldrich, USA), and 20 *μ*l of xanthine oxidase (0.90 mU/ml) (Sigma-Aldrich, USA). The optical density at 415 nm was recorded. SOD enzyme (Sigma-Aldrich, USA) activities at the concentrations of 0-25 units/ml were used as standard, and the results were expressed as units/mg protein.

Catalase activity was assessed based on the ability of the enzyme to break down H_2_O_2_. In brief, the reaction mixture containing 50 *μ*l of 30 mM hydrogen peroxide (in 50 mM phosphate buffer, pH 7.0) (BDH Chemicals Ltd., UK), 25 *μ*l of 5 M H_2_SO_4_ (Sigma-Aldrich, USA), and 150 *μ*l of 5 mM KMnO_4_ (Sigma-Aldrich, USA) was mixed with 10 *μ*l of sample. After mixing thoroughly, an absorbance at 490 nm was measured [50]. CAT enzyme (Sigma-Aldrich, USA) at the concentration range between 10 and 100 units/ml was used as standard, and the result was expressed as units/mg protein.

Glutathione peroxidase activity was also assessed. In brief, 20 *μ*l of the sample solution was mixed with the reaction mixture consisting of 10 *μ*l of 1 mM dithiothreitol (DTT) (Sigma-Aldrich, USA) and 10 mM monosodium phosphate (NaH_2_PO_4_) in DW, 100 *μ*l of 1 mM sodium azide (Sigma-Aldrich, USA) in 40 mM potassium phosphate buffer (pH 7.0), 10 *μ*l of 50 mM glutathione (Sigma-Aldrich, USA) solution, and 100 *μ*l of 30% hydrogen peroxide (BDH Chemicals Ltd., UK) and incubated at room temperature for 10 minutes. Then, 10 *μ*l of 10 mM DTNB (5,5-dithiobis-2-nitrobenzoic acid) (Sigma-Aldrich, USA) was added and an absorbance at 412 nm was recorded [51]. The standard calibration curve was prepared by using the GSH-Px enzyme (Sigma-Aldrich, USA) at the concentration range of 1-5 units/ml. GSH-Px activity was expressed as units/mg protein.

### 2.10. Western Blotting Analysis

Adipose tissue was homogenized and lysed in RIPA (radioimmunoprecipitation assay) buffer (1 : 5, *w*/*v*) (Cell Signaling Technology, USA) containing 20 mM Tris-HCl (pH 7.5), 150 mM NaCl, 1 mM Na_2_EDTA, 1 mM EGTA, 1% NP-40, 1% sodium deoxycholate, 2.5 mM sodium pyrophosphate, 1 mM beta-glycerophosphate, 1 mM Na_3_VO_4_, 1 *μ*g/ml leupeptin, and 1 mM phenylmethanesulfonyl fluoride (PMSF) (Cell Signaling Technology, USA). The tissue homogenate supernatant of the middle layer of fat samples was isolated after the 12,000 × *g* centrifugation at 4°C for 10 minutes. Protein concentration was determined by using a Thermo Scientific NanoDrop 2000c spectrophotometer (Thermo Fisher Scientific, Wilmington, Delaware, USA). In addition, eighty micrograms of tissue lysates was adjusted to an appropriate concentration by using Tris-Glycine SDS-PAGE loading buffer and heated at 95°C for 10 minutes. The protein in the tissue sample was isolated via sodium dodecyl sulfate-polyacrylamide gel electrophoresis (SDS-PAGE) by loading 20 *μ*l of the tissue sample on SDS-polyacrylamide gel. Then, the separated bands were transferred to a nitrocellulose membrane, washed with 0.05% TBS-T, and incubated in blocking buffer (5% skim milk in 0.1% TBS-T) at room temperature for 1 hour. After the blocking process, the nitrocellulose membrane was incubated with anti-PPAR gamma (Abcam, UK; dilution 1 : 1000), anti-NF-*κ*B p65 (Cell Signaling Technology, USA; dilution 1 : 500), anti-TNF-*α* (Cell Signaling Technology, USA; dilution 1 : 500), and anti-*β*-actin (Cell Signaling Technology, USA; dilution 1 : 1000) antibodies at 4°C, overnight. Following the incubation period, the nitrocellulose membrane was rinsed with TBS-T (0.05%) again and incubated with anti-rabbit IgG, HRP-linked antibody (Cell Signaling Technology, USA; dilution 1 : 2000) at room temperature for 1 hour. The bands were visualized and quantitated by using the ECL detection systems (GE Healthcare) and LAS-4000 luminescent image analyzer (GE Healthcare). Band intensities were measured for statistical analysis using ImageQuant TL v.7.0 image analysis software (GE Healthcare). The expression was normalized using anti-*β*-actin. Data were presented as a relative density to the control normal diet group.

### 2.11. Statistical Analysis

All data are expressed as mean ± standard error of mean (SEM). Statistical significance was evaluated by using one-way analysis of variance (ANOVA), followed by Duncan's post hoc test. Student's *t* test was used for comparison of the means for two groups. Statistical significance was evaluated at *p* values <0.05. All statistical data analyses were performed using SPSS version 21.0 (IBM Corp. Released 2012. IBM SPSS Statistics for Windows).

## 3. Results

### 3.1. Assessment of Phenolic Compounds and Biological Activities

**Figure 1:**
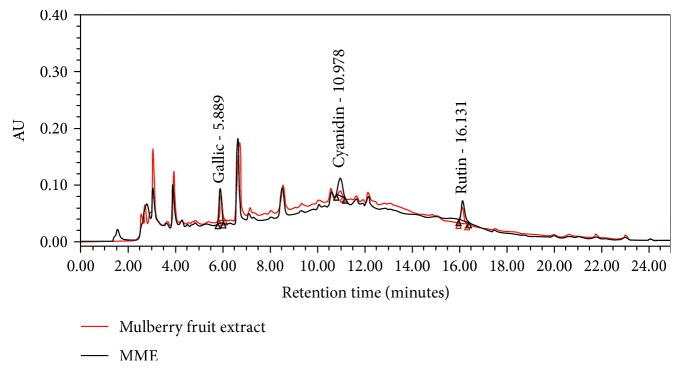
Fingerprint chromatogram of mulberry fruit extract (ME) and encapsulated mulberry fruit extract.

Table 1 shows that the contents of total phenolic compounds and flavonoid contents of the encapsulated mulberry fruit extract (MME) were 103.89 ± 13.08 mg GAE/mg sample extract and 26.56 ± 1.26 *μ*g quercetin/mg sample extract, respectively, whereas the contents of the substances just mentioned in mulberry fruit extract were 80.00 ± 0.98 mg GAE/mg sample extract and 8.89 ± 0.13 *μ*g quercetin/mg sample extract. The flavonoid content in MME was significantly higher than that in mulberry fruit extract (*p* value = 0.001). Phenolic compositions were identified using HPLC with UV-visible detection, and data are shown in Table 1 and Figure 1. It was found that the microencapsulated mulberry fruit extract (MME) at 200 milligrams contained 293.62 ± 4.90 *μ*g cyanidin 3-glucoside, 9.08 ± 0.09 *μ*g gallic acid, and 243.51 ± 5.88 *μ*g quercetin-3-O-rutinoside, whereas the mulberry fruit extract at 200 milligram contained 253.04 ± 3.92 *μ*g cyanidin-3-glucoside, 10.81 ± 0.29 *μ*g gallic acid, and 265.84 ± 17.66 *μ*g quercetin-3-O-rutinoside. No significant differences in gallic acid and quercetin-3-O-rutinoside contents between MME and mulberry fruit extract fruit extract were observed. Interestingly, the content of cyanidin-3-glucoside in MME was significantly higher than that in mulberry fruit extract (*p* value = 0.045). Since metabolic syndrome is comprised of the disorders of obesity, diabetes, hypertension, and dyslipidemia, the effects of the mulberry fruit extract and MME on the enzymes playing important roles on the mentioned conditions were also investigated and results are shown in Table 1. Regarding the suppression effects of the important enzymes in carbohydrate metabolism, including *α*-amylase, *α*-glucosidase, and aldose reductase, it was found that EC_50_ values of MME were 0.28 ± 0.002, 0.57 ± 0.04, and 0.14 ± 0.02 mg/ml, respectively, whereas those in mulberry fruit extract were 0.30 ± 0.13, 0.62 ± 0.06, and 0.35 ± 0.05 mg/ml. When compared to mulberry fruit extract, MME exerted a better effect on the suppression of aldose reductase activity (*p* value = 0.016). The suppression effects of the mulberry fruit extract and MME on ACE, lipase, and COX-2 activities were also assessed. EC_50_ values of the suppression effects of ACE, lipase, and COX-2 of MME were 0.08 ± 0.02, 0.34 ± 0.002, and 0.59 ± 0.04 whereas those of the mulberry fruit extract were 0.18 ± 0.01, 0.31 ± 0.01, and 1.36 ± 0.08 mg/ml, respectively. MME showed the significant potent suppression effects of ACE and COX-2 activities than those in mulberry fruit extract (*p* value = 0.019 and 0.006, respectively). In addition to the enzymes mentioned earlier, the pathophysiology of MetS also involved oxidative stress and inflammation. Therefore, the effects of MME on antioxidant were also explored. EC_50_ values of antioxidant activity assessed via DPPH, FRAP, and ABTS of MME were 0.04 ± 0.01, 601.91 ± 23.13, and 0.44 ± 0.02 mg/ml, respectively. EC_50_ values of antioxidant via the methods just mentioned on mulberry fruit extract were 0.43 ± 0.05, 560 ± 36.14, and 0.67 ± 0.03 mg/ml. The antioxidant activities assessed via DPPH and ABTS of MME were significantly better than those in the mulberry fruit extract (*p* value = 0.016 and 0.048, respectively).

### 3.2. Metabolic Parameter Changes

#### 3.2.1. Effect on Food Intake, Energy Intake, and Body Fat

Table 2 shows that HCHF diet failed to produce significant changes in food intake, but it significantly enhanced energy intake in both normal and OVX rats (*p* value < 0.05 all; compared to normal rats which received normal diet and vehicle). In addition, HCHF diet also increased the weights of abdominal fat (*p* value<0.001 all; compared to normal rats which received normal diet and vehicle). Isoflavone, L-carnitine, and MME at a dose of 250 mg/kg BW could attenuate the increase in abdominal fat (*p* value < 0.05, 0.001, and 0.05, respectively; compared to OVX rats which received HCHF and vehicle) in OVX rats which received HCHF diet.

#### 3.2.2. Effect on Body Weight Gain

The effects of MME on the metabolic parameters are shown in Table 3. It was found that HCHF significantly increased the percent of body weight gain both in normal and in OVX rats (*p* = 0.016 and 0.033, respectively; compared to normal rats, which received normal diet and vehicle). Both isoflavone and L-carnitine, which served as positive control in this study, could counteract the increase in the percent of body weight gain induced by HCHF in ovariectomized rats (*p* value = 0.009 and 0.006, respectively; compared to OVX rats, which received HCHF and vehicle). OVX rats which received MME at doses of 10 and 250 mg/kg BW also showed the significant reduction in percent of body weight gain (*p* value = 0.048 and 0.016, respectively; compared to OVX rats, which received HCHF and vehicle).

### 3.3. Effect on Lipid Profiles and Atherogenic Index

Serum lipid profiles, including total cholesterol, triglycerides, HDL-C, and LDL-C, are shown in Table 3. The current data demonstrated that HCHF diet treatment for 4 weeks significantly decreased HDL-C but increased LDL-C and failed to produce the significant changes of total cholesterol and triglycerides (*p* value < 0.001 and 0.033, respectively, compared to normal rats, which received normal diet and vehicle) in normal rats. However, HCHF diet could increase total cholesterol, triglycerides, and LDL-C but decreased HDL-C in OVX rats at this duration of treatment (*p* value < 0.001, *p* value < 0.001, *p* value = 0.005, and *p* value < 0.001, respectively, compared to normal rats which received normal diet and vehicle). OVX rats, which were treated with HCHF diet and received isoflavone, showed the significant decrease in total cholesterol, triglycerides, and LDL-C (*p* value < 0.001, *p* value = 0.003, and *p* value < 0.001, respectively, compared to OVX rats which received HCHF and vehicle) but failed to show the significant change in HDL-C. OVX rats, which received HCHF diet and received L-carnitine, significantly decreased total cholesterol, triglycerides, and LDL-C but increased decreased HDL-C in OVX rats at this duration of treatment (*p* value <0.001, *p* value < 0.001, *p* value = 0.023, and *p* value < 0.001, respectively, compared to OVX rats which received HCHF and vehicle). At the 4-week intervention period, all doses of MME used in this study also significantly decreased total cholesterol (*p* value = 0.001, *p* value < 0.001, and *p* value < 0.001, respectively, compared to OVX rats, which received HCHF and vehicle), triglycerides (*p* value = 0.046, *p* value = 0.006, and *p* value = 0.001, respectively, compared to OVX rats which received HCHF and vehicle), and LDL-C (*p* value <0.001, *p* value = 0.001, and *p* value = 0.036, respectively, compared to OVX rats which received HCHF and vehicle) but increased HDL-C (*p* value = 0.003, *p* value = 0.001, and *p* value < 0.001, respectively, compared to OVX rats, which received HCHF and vehicle). When the treatment was prolonged to 8 weeks, HCHF diet still failed to change total cholesterol and triglyceride levels. The decreased HDL-C was still observed (*p* value < 0.001; compared to normal rats, which received normal diet) whereas the increased LDL-C did not present in normal rats anymore. In OVX rats, HCHF diet could increase total cholesterol, triglycerides, and LDL-C but increased HDL-C (*p* value = 0.001, *p* value < 0.001, *p*-value = 0.037, and *p* value < 0.001, respectively, compared to normal rats which received normal diet and vehicle). At the 8-week intervention period, OVX rats which received HCHF diet and isoflavone, L-carnitine, and all doses of MME significantly mitigated the elevation of total cholesterol (*p* value < 0.001, *p* value = 0.012, *p* value = 0.007, *p* value = 0.014, and *p* value = 0.013, respectively, compared to OVX rats which received HCHF and vehicle), triglycerides (*p* value = 0.008, *p* value < 0.001, *p* value = 0.048, *p* value = 0.022, and *p* value = 0.005, respectively, compared to OVX rats which received HCHF and vehicle) and LDL-C (*p* value < 0.001 all; compared to OVX rats which received HCHF and vehicle). The reduction of HDL-C in OVX rats which received HCHF diet was also attenuated by L-carnitine and all doses of MME (*p* value < 0.001, *p* value < 0.001, *p* value = 0.004, and *p* value = 0.009, respectively, compared to OVX rats which received HCHF and vehicle).

Atherogenic index (AI), one of the parameters used for indicating cardiovascular risk, was also investigated. The current data showed that at 4 and 8 weeks of the study period, HCHF diet could increase AI in both normal and OVX rats (*p* value = 0.003 and *p* value = 0.005, respectively, and *p*-value < 0.001 all, compared to normal rats which received normal diet and vehicle). At 4 weeks of intervention, the elevation of AI in OVX rats which received HCHF was attenuated by L-carnitine and MME at doses of 10, 50, and 250 mg/kg BW (*p* value = 0.041, *p* value = 0.049, *p* value = 0.020, and *p* value = 0.038, respectively, compared to OVX rats which received HCHF and vehicle). When the treatments were prolonged to 8 weeks, the improved AI in OVX rats with HCHF diet treatment was still observed in those which received isoflavone, L-carnitine, and all doses of MME (*p* value = 0.020, *p* value < 0.001, *p* value < 0.001, *p* value < 0.001, and *p* value < 0.001, respectively, compared to OVX rats, which received HCHF and vehicle) as shown in Table 3.

### 3.4. Changes of Fasting Blood Sugar and Glucose Area under the Curve during Oral Glucose Tolerance Test (OGTT)

It was found that HCHF diet increased fasting blood sugar in normal rats at 8 weeks and in OVX rats at both 4 and 8 weeks of intervention (*p* value < 0.001, *p* value = 0.026, and *p* value = 0.018, respectively, compared to normal rats which received normal diet and vehicle). After 4 weeks of treatment, OVX rats which received MME at doses of 10, 50, and 250 mg/kg BW significantly mitigated the elevation of fasting blood sugar induced by HCHF diet in OVX rats (*p* value = 0.045, *p* value = 0.001, and *p* value = 0.017, respectively, compared to OVX rats which received HCHF and vehicle). However, at 8 weeks of intervention, the significant reduction in blood sugar was observed only in OVX rats which received medium and high doses of MME treatment (*p* value = 0.043 and *p* value = 0.045, respectively, compared to OVX rats which received HCHF and vehicle) as shown in Table 3.

Glucose areas under the curve during the oral glucose tolerance test at 4 and 8 weeks of the treatment period are also shown in Table 3. The results showed that normal rats and OVX rats which received HCHF diet and vehicle significantly increased the plasma glucose area under the curve at 4 weeks and 8 weeks of experiments (*p* value = 0.008 and *p* value = 0.002, respectively, and *p* value = 0.032 and *p*-value <0.001, respectively, compared to normal rats which received normal diet and vehicle group). At 4 weeks of the study period, only OVX rats, which were fed with HCHF diet and received MME at a dose of 50 mg/kg BW, significantly decreased the plasma glucose area under the curve (*p* value = 0.031, compared to OVX rats which received HCHF and vehicle). When the treatment was prolonged, isoflavone, L-carnitine, and all doses of MME could increase the plasma glucose area under the curve in OVX rats with HCHF diet (*p* value = 0.009, *p* value = 0.022, *p* value = 0.007, *p* value = 0.006, and *p* value = 0.042, respectively, compared to OVX rats which received HCHF and vehicle).

### 3.5. The Suppression of Angiotensin-Converting Enzyme (ACE) Activity

Table 3 shows that HCHF diet significantly increased the activity of ACE (*p* value < 0.001 all, compared to normal rats which received normal diet and vehicle). This elevation was mitigated by isoflavone, L-carnitine, and MME at doses of 10, 50, and 250 mg/kg BW (*p* value = 0.005, *p* value = 0.001, *p* value = 0.016, *p* value <0.001, and *p* value <0.001, respectively, compared to OVX rats which received HCHF and vehicle).

### 3.6. Changes of Serum Estradiol

Table 4 shows that HCHF diet failed to produce the significant change of serum estradiol in female rats. However, bilateral ovariectomy significantly decreased serum estradiol in female rats fed with HCHF diet (*p* value<0.05, compared to control rats which received normal diet and vehicle or ND + vehicle; *p* value<0.01; compared to normal rats which received HCHF diet and vehicle). Isoflavone, L-carnitine, and all doses of MME failed to produce the significant changes of estradiol level in OVX rats which received HCHF diet.

### 3.7. Changes in Adiposity

**Figure 2:**
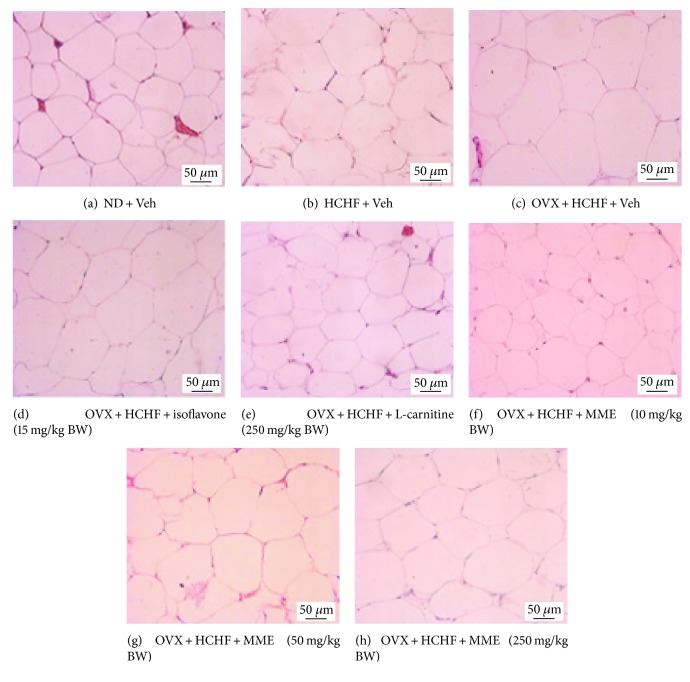
Representative of white adipose tissue at the gonadal area stained with hematoxylin and eosin (H&E) (40X) of rats with various treatments. (a) normal diet (ND) + vehicle, (b) HCHF + vehicle, (c) OVX-HCHF diet + vehicle, (d) OVX-HCHF diet + isoflavone 15 mg/kg BW, (e) OVX-HCHF diet + L-carnitine 250 mg/kg BW, (f) OVX-HCHF diet + MME 10 mg/kg BW, (g) OVX-HCHF diet + MME 50 mg/kg BW, and (h) OVX-HCHF diet + MME 250 mg/kg BW.

**Figure 3:**
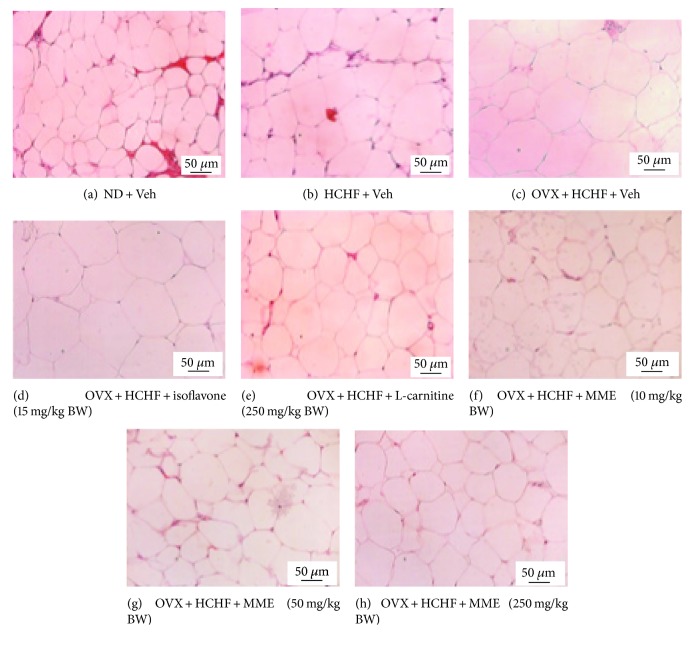
Representative of white adipose tissue at the mesenteric area stained with hematoxylin and eosin (H&E) (40X) of rats with various treatments. (a) Normal diet (ND) + vehicle, (b) HCHF + vehicle, (c) OVX-HCHF diet + vehicle, (d) OVX-HCHF diet + isoflavone 15 mg/kg BW, (e) OVX-HCHF diet + L-carnitine 250 mg/kg BW, (f) OVX-HCHF diet + MME 10 mg/kg BW, (g) OVX-HCHF diet + MME 50 mg/kg BW, and (h) OVX-HCHF diet + MME 250 mg/kg BW.

**Figure 4:**
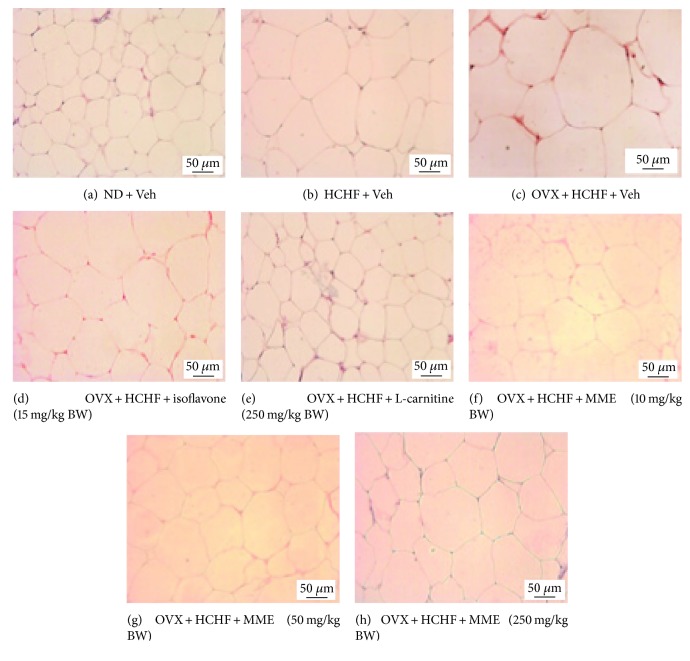
Representative of white adipose tissue at the retroperitoneal area stained with hematoxylin and eosin (H&E) (40x) of rats with various treatments. (a) Normal diet (ND) + vehicle, (b) HCHF + vehicle, (c) OVX-HCHF diet + vehicle, (d) OVX-HCHF diet + isoflavone 15 mg/kg BW, (e) OVX-HCHF diet + L-carnitine 250 mg/kg BW, (f) OVX-HCHF diet + MME 10 mg/kg BW, (g) OVX-HCHF diet + MME 50 mg/kg BW, and (h) OVX-HCHF diet + MME 250 mg/kg BW.

**Figure 5:**
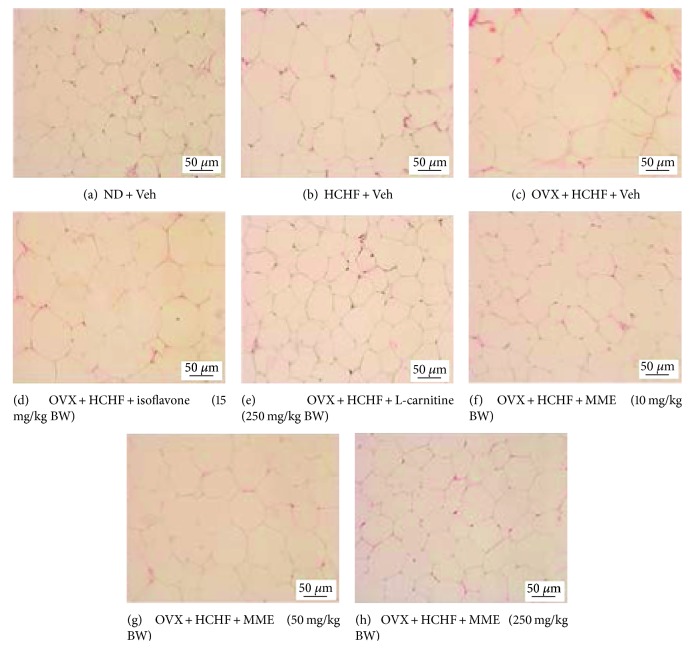
Representative of white adipose tissue at the subcutaneous area stained with hematoxylin and eosin (H&E) (40x) of rats with various treatments. (a) Normal diet (ND) + vehicle, (b) HCHF + vehicle, (c) OVX-HCHF diet + vehicle, (d) OVX-HCHF diet + isoflavone 15 mg/kg BW, (e) OVX-HCHF diet + L-carnitine 250 mg/kg BW, (f) OVX-HCHF diet + MME 10 mg/kg BW, (g) OVX-HCHF diet + MME 50 mg/kg BW, and (h) OVX-HCHF diet + MME 250 mg/kg BW.

**Figure 6:**
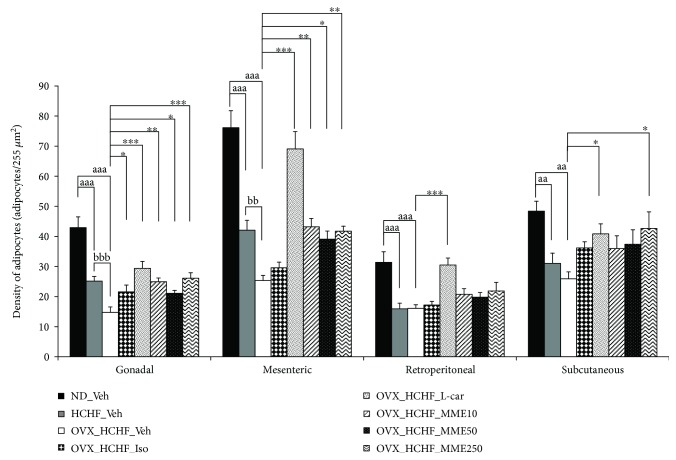
Density of adipocytes at various regions of OVX rats with HCHF diet which received various treatments. Data are presented as mean ± SEM (*n* = 6/group). ^aa, aaa^*p* value < 0.01 and 0.001, respectively, compared to control rats which received normal diet and vehicle, ^bb, bbb^*p* value < 0.01 and 0.001, respectively, compared to normal rats which received HCHF diet and vehicle and ^∗^, ^∗∗^, ^∗∗∗^*p*-value <0.05, 0.01, and 0.001, respectively, compared to OVX rats which received HCHF and vehicle. ND: normal diet; HCHF: high-carbohydrate high-fat diet; OVX-HCHF: ovariectomized plus high-carbohydrate high-fat diet; Iso: isoflavone at a dose of 15 mg/kg BW; L-car: L-carnitine at a dose of 250 mg/kg BW; MME10, 50, and 250, the microencapsulated mulberry fruit extract at doses of 10, 50, and 250 mg/kg BW, respectively.

**Figure 7:**
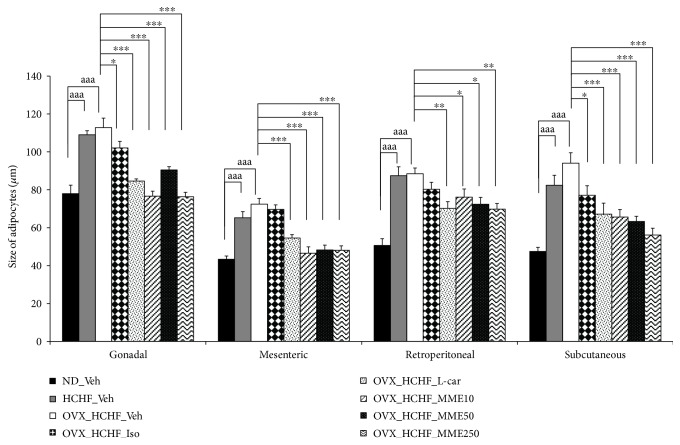
Size of adipocytes at various regions of OVX rats with HCHF diet which received various treatments. Data are presented as mean ± SEM (*n* = 6/group). ^aaa^*p* value < 0.001; compared to control rats which received normal diet and vehicle and ^∗^, ^∗∗^, ^∗∗∗^*p* value < 0.05, 0.01, and 0.001, respectively, compared to OVX rats which received HCHF and vehicle. ND: normal diet; HCHF: high-carbohydrate high-fat diet; OVX-HCHF: ovariectomized plus high-carbohydrate high-fat diet; Iso: the isoflavone at dose of 15 mg/kg BW; L-car: L-carnitine at a dose of 250 mg/kg BW; MME10, 50, and 250: the microencapsulated mulberry fruit extract at doses of 10, 50, and 250 mg/kg BW, respectively.

Figures 2–7 show the effect of MME on density and size of adipocyte in gonadal, mesenteric, retroperitoneal, and subcutaneous areas. The decreased density of adipocyte cells was observed in gonadal, mesenteric, retroperitoneal, and subcutaneous areas of both normal and OVX rats, which received HCHF diet (*p* value < 0.001 all, *p* value < 0.001 all, *p* value <0.001 all, and *p*-value = 0.006 and 0.001, respectively, compared to normal rats which received normal diet and vehicle). Isoflavone could increase adipocyte density only in the gonadal area of OVX rats fed with HCHF diet (*p* value = 0.042; compared to OVX rats which received HCHF and vehicle) whereas L-carnitine could increase adipocyte density in all areas mentioned above (*p* value < 0.001, *p* value < 0.001, *p* value <0.001, and *p* value = 0.032, respectively, compared to OVX rats which received HCHF and vehicle). Both low and medium doses of MME could increase adipocyte density in gonadal and mesenteric areas (*p* value = 0.001 and *p* value = 0.003; *p* value = 0.038 and *p* value = 0.017; compared to OVX rats which received HCHF and vehicle) whereas the high dose of MME produced the significant elevation of adipocyte density in gonadal, mesenteric, and subcutaneous areas (*p* value < 0.001, *p* value = 0.005, and *p* value = 0.010, respectively, compared to OVX rats which received HCHF and vehicle) as shown in Figure 6.

In addition to the effect on adipocyte density, the effect of MME on adipocyte size was also determined.  Figure 7 shows that HCHF significantly increased adipocyte size in all areas investigated in this study (*p* value < 0.001 all, compared to normal rats, which received normal diet and vehicle). Isoflavone significantly mitigated the increase in adipocyte size in gonadal and subcutaneous areas induced by HCHF diet (*p* value = 0.029 and 0.021, respectively; compared to OVX rats which received HCHF and vehicle) whereas L-carnitine and MME at doses of 10, 50, and 250 mg/kg BW produced a significant reduction in adipocyte size in gonadal, mesenteric, retroperitoneal, and subcutaneous areas of OVX rats, which were fed with HCHF diet (*p* value < 0.001, *p* value < 0.001, *p* value = 0.005, and *p* value <0.001, respectively; *p*-value < 0.001, *p* value < 0.001, *p* value = 0.042, and *p*-value <0.001, respectively; *p* value < 0.001, *p* value < 0.001, *p* value = 0.012, and *p* value <0.001, respectively; and *p* value < 0.001, *p* value < 0.001, *p* value = 0.003, and *p* value < 0.001, respectively, compared to OVX rats which received HCHF and vehicle).  Table 5 shows that both normal and OVX rats which received HCHF diet also increased the adiposity index (*p* value < 0.001 all; compared to normal rats which received normal diet and vehicle). However, this elevation was mitigated by isoflavone, L-carnitine, and MME at a dose of 250 mg/kg BW when compared to OVX rats which received HCHF and vehicle (*p* value = 0.020, *p* value < 0.001 and *p* value = 0.016, respectively, compared to OVX rats which received HCHF and vehicle).

### 3.8. Effect of MME on the Alteration of Oxidative Stress Status

Table 6 shows that HCHF diet significantly increased the MDA level in adipose tissue of both normal and OVX rats (*p* value < 0.001 all; compared to control rats which received normal diet and vehicle). Isoflavone, L-carnitine, and MME at doses of 10, 50, and 250 mg/kg significantly mitigated the elevation of the MDA level in adipose tissue of OVX rats which were fed with HCHF diet (*p* value = 0.015, *p* value < 0.001, *p* value < 0.001, *p* value = 0.005, and *p* value < 0.001, respectively, compared to OVX rats which received HCHF and vehicle). HCHF diet also produced the significant reduction in SOD, CAT, and GSH-Px activities in adipose tissues of normal and OVX rats (*p* value <0.001 all; *p* value = 0.022 and 0.001, respectively; and *p*-value = 0.047 and 0.005, respectively, compared to control rats which received normal diet and vehicle). Isoflavone could mitigate the decrease in SOD and GSH-Px activity (*p* value < 0.001 and *p* value = 0.036, respectively, compared to OVX rats which received HCHF and vehicle) while L-carnitine significantly increased in SOD activity (*p* value = 0.012, compared to OVX rats which received HCHF and vehicle). Interestingly, MME at doses of 10, 50, and 250 mg/kg could increase SOD (*p* value <0.001, *p* value = 0.041, and *p* value = 0.007, respectively, compared to OVX rats which received HCHF and vehicle), CAT (*p* value = 0.006, *p* value = 0.027, and *p* value = 0.016, respectively, compared to OVX rats which received HCHF and vehicle), and GSH-Px (*p* value = 0.020, *p* value = 0.033, and *p* value = 0.049, respectively, compared to OVX rats which received HCHF and vehicle) activities in adipose tissue of OVX rats which were fed with HCHF diet.

### 3.9. Changes in Inflammatory Mediators

**Figure 8:**
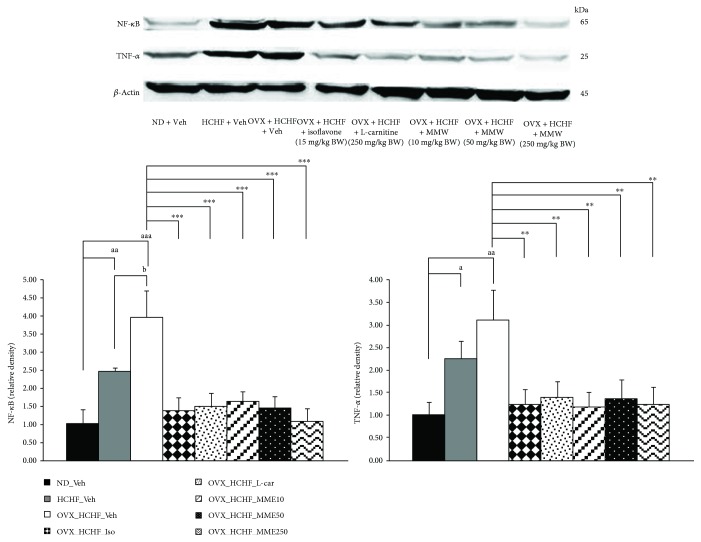
The relative density of NF-*κ*B and TNF-*α* in adipose tissue of OVX rats with HCHF diet which received various treatments. The levels of *β*-actin were normalized against the level of NF-*κ*B and TNF-*α*. The relative density of NF-*κ*B and TNF-*α* levels were calculated against those of control normal diet plus vehicle rats. Data are presented as mean ± SEM (*n* = 6/group). ^a, aa, aaa^*p* value < 0.05, 0.01, and 0.001, respectively, compared to control rats, which received normal diet and vehicle, ^b^*p* value < 0.05, compared to normal rats, which received HCHF diet and vehicle and ^∗∗^, ^∗∗∗^*p* value <0.01 and 0.001, respectively, compared to OVX rats which received HCHF and vehicle. ND: normal diet; HCHF: high-carbohydrate high-fat diet; OVX-HCHF: ovariectomized plus high-carbohydrate high-fat diet; Iso: the isoflavone at dose of 15 mg/kg BW; L-car: L-carnitine at a dose of 250 mg/kg BW; MME10, 50, and 250: the microencapsulated mulberry fruit extract at doses of 10, 50, and 250 mg/kg BW, respectively.

Figure 8 showed that both normal and OVX rats which were fed with HCHF diet increased NF-*κ*B (*p* value = 0.008 and *p* value < 0.001, respectively, compared to normal rats which received normal diet and vehicle) and TNF-*α* (*p* value = 0.035 and *p* value = 0.001, respectively, compared to normal rats which received normal diet and vehicle) in adipose tissue. However, these changes were mitigated by isoflavone, L-carnitine, and all doses of MME (*p* value < 0.001 all; *p* value = 0.004, *p* value = 0.008, *p* value = 0.002, *p* value = 0.005, and *p*-value = 0.003, respectively, compared to OVX rats which received HCHF and vehicle).

### 3.10. Effect of MME on Peroxisome Proliferator-Activated Receptor Gamma (PPAR-*γ*)

**Figure 9:**
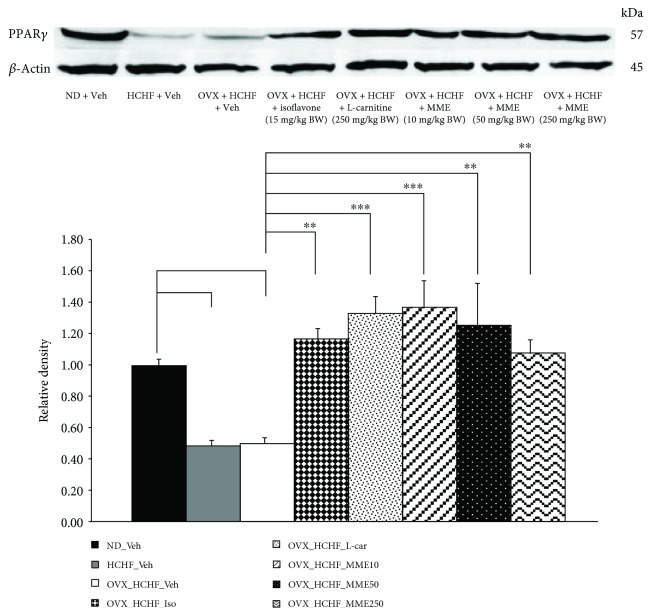
The relative density of PPAR-*γ* in adipose tissue of OVX rats with HCHF diet which received various treatments. The levels of *β*-actin were normalized against the level of PPAR-*γ*. Their relative density of PPAR-*γ* levels were calculated against those of control normal diet plus vehicle rats. Data are presented as mean ± SEM (*n* = 6/group). ^a^*p* value < 0.05, compared to control rats which received normal diet and vehicle and ^∗∗^ and ^∗∗∗^*p* value < 0.01 and 0.001, respectively, compared to OVX rats which received HCHF and vehicle. ND: normal diet; HCHF: high-carbohydrate high-fat diet; OVX-HCHF: ovariectomized plus high-carbohydrate high-fat diet; Iso: the isoflavone at a dose of 15 mg/kg BW; L-car: the L-carnitine at a dose of 250 mg/kg BW; MME10, 50, and 250, the microencapsulated mulberry fruit extract at doses of 10, 50, and 250 mg/kg BW, respectively.

The effect of MME on PPAR-*γ*, a factor playing an important role on adipogenesis and lipotoxicity was also investigated, and results are shown in Figure 9. It was found that normal rats and OVX rats which received HCHF diet significantly decreased the relative density of PPAR-*γ* in adipose tissues (*p* value = 0.036 and *p* value = 0.041, respectively, compared to control rats which received normal diet and vehicle). The results showed that isoflavone, L-carnitine, and MME at all doses used in this study significantly increased the relative density of PPAR-*γ* in adipose tissues (*p* value = 0.008, *p* value <0.001, *p* = 0.007, *p* value = 0.001, and *p* value = 0.012, respectively, compared to OVX rats which received HCHF and vehicle).

## 4. Discussion

This study demonstrated that the encapsulated mulberry fruit extract contained abundant polyphenol compounds. HPLC analysis showed that phenolic compounds, such as cyanidin 3-glucoside, quercetin 3-O-rutinoside, and gallic acid were presented in the MME extract. In addition, it also possessed numerous biological activities related with the pathophysiology of metabolic syndrome. Therefore, it was worth moving forwards to in vivo experiment in the preclinical phase. Data obtained from the animal model clearly demonstrated that MME could decrease total cholesterol, triglycerides, LDL-C, and AI-index but increased HDL-C. It also decreased fasting blood sugar, glucose area under the curve during OGTT, and ACE activity in serum. The reduction of various parameters, including the density and size of adipocyte cells in both visceral and subcutaneous areas, adiposity index, MDA level, TNF-*α*, and NF-KB together with the increase in SOD, CAT, and GSH-Px activities and PPAR-*γ* in adipose tissue were also observed in OVX rats which were induced metabolic syndrome by HCHF diet and received MME. However, no significant changes of serum estradiol, food intake, and energy intake were observed.

Since rats fed with HCHF diet produced numerous disorders that mimicked metabolic syndrome condition [52], it has been used as experimental animal in this study. The current results were in agreement with previous findings, which demonstrated that HCHF induced disorders of metabolic parameters, such as body weight, visceral fat mass [53], plasma total cholesterol and triglyceride levels [54], MDA level, and inflammation in adipose tissue [5, 54] in OVX rats more than that in normal rats. This was also corresponding with the previous study, which demonstrated that postmenopausal condition increased the vulnerability to various insults [4].

Accumulative lines of evidence during the last decade have demonstrated that PPAR-*γ* plays an important role on the regulation of various genes playing important roles on the regulation of the intermediary metabolism of glucose, lipid, homeostasis, and adipogenesis, especially in an insulin-sensitive adipocyte [55, 56]. It has been reported that PPAR-*γ* stimulation could induce apoptosis of old or hypertrophy adipocyte [57–59], decreased triglyceride [58], LDL [59], and increased HDL [58]. In addition, the stimulation of PPAR-*γ* also plays a crucial role on glucose metabolism. It can increase the expression and translocation of the glucose transporter resulting in the increase in glucose uptake into adipocyte and muscle cells resulting in the reduction of blood sugar [60]. The increased PPAR-*γ* expression also improves the oral glucose tolerance test [61]. The stimulation of PPAR-*γ* not only exerts its influence on glucose and lipid metabolism but also suppresses gene expression of the angiotensin-converting enzyme (ACE) [62]. PPAR-*γ* also exerts anti-inflammatory effects via the inhibitory effect on the production of inflammatory cytokines, such as tumor necrosis factor-*α* in the monocytes and nuclear factor kappa B (NF-*κ*B) [63]. In addition, it has been reported that during acute response of metabolic syndrome, the expression of PPAR-*γ* decreases resulting in the increased production of free radicals and oxidative stress [64].

The data obtained from this study had revealed that HCHF diet decreased PPAR-*γ* expression giving rise to the reduction in adipocyte density. The increase in the lipid profile induced by HCHF also increased in the lipid uptake and storage in adipocyte giving rise to adipocyte hypertrophy. Both adipocyte hypertrophy and the increased fat pad in both subcutaneous and visceral regions induced the increase in body weight gain and the elevations of TNF-*α*, NF-*κ*B, hyperglycemia, and poor response of the glucose tolerance test. It has been reported that excess abdominal fat contributes to dyslipidemia, hypertension, and insulin resistance [65]. However, subcutaneous fat also plays a role on glycemic control [66]. The elevation of lipid profiles observed in this study also induced the increased AI whereas the increase in adipocyte gave rise to the increase in adiposity index. The reduction in PPAR-*γ* expression also induced the elevation of ACE and MDA levels. In addition, the increase in NF-*κ*B also enhanced the MDA level [67]. The elevation of the MDA level also occurred partly via the reduction of antioxidant enzymes, such as SOD, CAT, and GSH-Px, induced by HCHF diet [68]. Interestingly, all changes mentioned earlier except the increase in retroperitoneal pad density could be mitigated by MME. MME could also decrease atherosclerosis risk as shown by the improved AI.

Based on the previous study that cyanidin is an agonist of PPAR-*γ* [69], we suggest that the positive modulation effect of MME observed in this study may be associated with the cyanidin substance presented in MME. Our data also demonstrate that when compared to soy isoflavone or genistein which is currently available in the market, MME improves the serum HDL level, atherogenic index, and oxidative stress status better than soy isoflavone does. In addition, MME treatment especially at low and medium doses for 8 weeks also increases glycemic control capability reflected by the reduction in plasma glucose AUC [70]. The possible explanation may be associated partly with the protection of active ingredients such as polyphenolic compound including cyanidins by a core material which is used for encapsulation [71, 72]. However, the improvement of most metabolic parameters induced by MME and L-carnitine shows no significant differences. Only the improvement of plasma glucose AUC of metabolic syndrome rats which received MME is better than L-carnitine. Therefore, this study is the first study to reveal that MME is the potential candidate for mitigating metabolic syndrome in menopausal women. Interestingly, the effectiveness of MME is better than soy isoflavone and it has better glycemic control than L-carnitine which is available in the market.

This study failed to show the dose-dependent study, because the observed parameters were under the influences of multiple factors so there is no simple linear relationship between concentrations of MME and the observed parameters. In addition, MME was the crude extract so it was possible that inactive ingredients might mask the effect of active ingredients.

## 5. Conclusions

**Figure 10:**
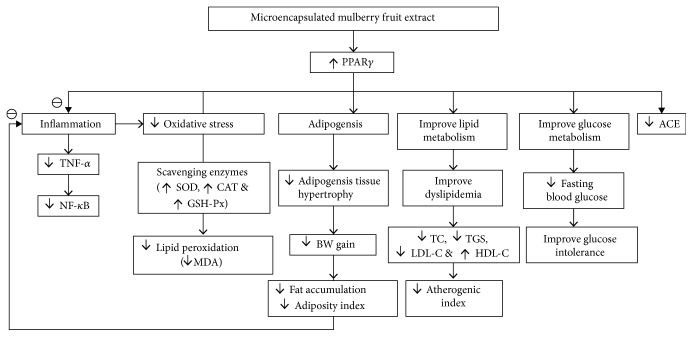
Schematic diagram showing the possible actions of encapsulated mulberry extract.

This study demonstrates that MME is the potential candidate for improving metabolic syndrome and its related complication, such as cardiovascular disorder by improving all metabolic parameters, inflammation, and oxidative stress status. The possible underlying mechanism may occur partly via the increased PPAR-*γ* expression and the elevations of main scavenger enzymes, such as SOD, CAT, and GSH-Px as shown in Figure 10. MME not only improves the metabolic syndrome condition but also decreases its complications, such as cardiovascular disorders. However, the clinical study to confirm this benefit is essential.

## Figures and Tables

**Table 1:** Phenolic compound content and biological activity of mulberry fruit extract (ME) and encapsulated mulberry fruit extract (MME).

Parameters	Units	ME-50% hydroalcoholic	MME	Standard reference
Total phenolic	mg GAE/mg extract	80.00 ± 0.98	103.89 ± 13.08	—
Total flavonoids	*μ*g quercetin/mg extract	8.89 ± 0.13	26.56±1.26^∗∗^	—
Cyanidin 3-glucoside	*μ*g Cyn-3-glu/200 mg extract	253.04 ± 3.92	293.62 ± 4.90^∗^	—
Quercetin 3-O-rutinoside (rutin)	*μ*g quercetin-3-O-rutinoside/200 mg extract	265.84 ± 17.66	243.51 ± 5.88	—
Gallic acid	*μ*g gallic/200 mg extract	10.81 ± 0.29	9.08 ± 0.09	—
Antioxidant activities				
DPPH	EC_50_ (mg/ml)	0.43 ± 0.05	0.04 ± 0.01^∗^	0.03 ± 0.01, ascorbic acid
FRAP	EC_50_ (mg/ml)	560.05 ± 36.14	601.91 ± 23.13	122.19 ± 12.82, ascorbic acid
ABTS	EC_50_ (mg/ml)	0.67 ± 0.03	0.44 ± 0.02^∗^	0.15 ± 0.001, Trolox
Antidiabetic markers				
*α*-Amylase inhibition	EC_50_ (mg/ml)	0.30 ± 0.13	0.28 ± 0.002	—
*α*-Glucosidase inhibition	EC_50_ (mg/ml)	0.62 ± 0.06	0.57 ± 0.04	—
Aldose reductase inhibition	EC_50_ (mg/ml)	0.35 ± 0.05	0.14 ± 0.02^∗^	0.004 ± 0.003, quercetin
Cardiovascular marker				
ACE inhibition	EC_50_ (mg/ml)	0.18 ± 0.01	0.08 ± 0.02^∗^	0.03 ± 0.01, Captopril
Obesity marker				
Lipase inhibition	EC_50_ (mg/ml)	0.31 ± 0.01	0.34 ± 0.002	0.002 ± 0.001, Orlistat
Inflammatory marker				
COX-2 inhibition	EC_50_ (mg/ml)	1.36 ± 0.08	0.59±0.04^∗∗^	0.02 ± 0.001, Indomethacin

Data are presented as mean ± SEM. Values are statistically significantly different by Student's *t* test compared between MME and mulberry fruit extract. (^∗^, ^∗∗^*p* < 0.05 and 0.01, respectively).

**Table 2:** The effect of various doses of MME on food intake and abdominal fat weight.

Treatment group	Food intake (g/day)	Energy intake (kcal/g)	Abdominal fat weight (g)
ND + vehicle	10.84 ± 0.16	43.58 ± 0.63	10.77 ± 0.73
HCHF + vehicle	10.93 ± 0.14	50.51 ± 0.63^a^	44.39 ± 7.68^aaa^
OVX + HCHF + vehicle	10.56 ± 0.12	49.76 ± 0.77^a^	58.17 ± 4.09^aaa^
OVX + HCHF + isoflavone 15 mg/kg BW	11.32 ± 0.57	53.51 ± 2.78	40.05 ± 3.94^∗^
OVX + HCHF + L-carnitine 250 mg/kg BW	9.73 ± 0.31	44.96 ± 1.41	26.77±3.94^∗∗∗^
OVX + HCHF + MME 10 mg/kg BW	11.21 ± 0.45	52.83 ± 2.17	43.57 ± 3.92
OVX + HCHF + MME 50 mg/kg BW	11.00 ± 0.24	51.82 ± 1.29	46.43 ± 5.38
OVX + HCHF + MME 250 mg/kg BW	10.46 ± 0.48	49.18 ± 2.00	38.68 ± 4.36^∗^

Data are presented as mean ± SEM (*n* = 6/group). ^a, aaa^*p* value < 0.05 and 0.001, respectively, compared to control rats which received normal diet and vehicle and ^∗^, ^∗∗∗^*p* value < 0.05 and 0.001, respectively, compared to OVX rats which received HCHF and vehicle. ND: normal diet; HCHF: high-carbohydrate high-fat diet; OVX + HCHF: ovariectomized rats which received high-carbohydrate high-fat diet; MME10, 50, and 250: an encapsulated mulberry fruits extract at doses of 10, 50, and 250 mg/kg BW, respectively.

**Table 3:** Changes of metabolic parameters of OVX rats with HCHF diet after the administration of ME and MME.

Parameters	ND + vehicle	HCHF + vehicle	OVX + HCHF + vehicle	OVX + HCHF + isoflavone15	OVX + HCHF + L-car250	OVX + HCHF + MME10	OVX + HCHF + MME50	OVX + HCHF + MME250
Body weight gain (%)	3.03 ± 1.43	9.32 ± 1.30^a^	8.96 ± 0.80^a^	1.53±2.78^∗∗^	1.12±2.00^∗∗^	3.73 ± 2.10^∗^	6.34 ± 0.57	2.15 ± 0.97^∗^
TC (mg/dl)								
4-week	138.92 ± 5.73	140.02 ± 4.21	240.93 ± 23.39^aaa,bbb^	139.03±6.65^∗∗∗^	167.77±6.48^∗∗∗^	191.29±9.52^∗∗^	154.15±6.89^∗∗∗^	174.86±6.87^∗∗∗^
8-week	134.64 ± 5.95	154.00 ± 4.33	193.58 ± 34.79^aa,b^	132.50±9.63^∗∗∗^	146.68 ± 1.88^∗^	146.12±1.51^∗∗^	152.65 ± 8.30^∗^	152.58 ± 3.96^∗^
Triglycerides (mg/dl)								
4-week	133.64 ± 1.72	132.43 ± 4.32	165.49 ± 2.10^aaa,bbb^	144.44±6.23^∗∗^	118.18±2.58^∗∗∗^	152.15 ± 4.75^∗^	145.58±2.76^∗∗^	142.64±1.16^∗∗^
8-week	131.88 ± 4.59	147.98 ± 10.53	167.59 ± 8.22^aaa,b^	142.35±3.59^∗∗^	127.56±4.69^∗∗∗^	146.99 ± 6.80^∗^	145.03 ± 1.87^∗^	141.25±1.85^∗∗^
HDL-C (mg/dl)								
4-week	43.45 ± 3.42	12.95 ± 5.17^aaa^	8.11 ± 1.46^aaa^	18.62 ± 0.85	20.84 ± 2.13^∗^	28.35±2.22^∗∗^	28.78±5.07^∗∗^	31.42±5.98^∗∗∗^
8-week	54.37 ± 2.63	9.30 ± 2.44^aaa^	7.22 ± 0.88^aaa^	11.19 ± 2.62	32.41±8.48^∗∗∗^	28.27±4.09^∗∗∗^	25.03±4.33^∗∗^	23.70±5.12^∗∗^
LDL-C (mg/dl)								
4-week	8.29 ± 0.36	16.32 ± 1.97^a^	20.20 ± 1.84^aa^	6.47±2.27^∗∗∗^	11.61 ± 5.06^∗^	5.91±1.97^∗∗∗^	8.04±0.68^∗∗^	11.68 ± 2.53^∗^
8-week	12.85 ± 1.37	17.32 ± 3.22	18.38 ± 0.34^a^	8.65±1.50^∗∗∗^	7.70±2.72^∗∗∗^	8.73±1.04^∗∗∗^	9.05±0.44^∗∗∗^	4.85±1.20^∗∗∗^
Atherogenic index								
4-week	3.26 ± 0.26	20.92 ± 6.57^aa^	20.63 ± 4.28^aa^	22.11 ± 7.45	8.40 ± 0.69^∗^	7.28 ± 0.97^∗^	5.72 ± 1.14^∗^	6.51 ± 0.85^∗^
8-week	2.61 ± 0.13	24.05 ± 4.67^aaa^	23.36 ± 3.15^aaa^	15.08 ± 3.81^∗^	6.02±1.28^∗∗∗^	6.20±0.93^∗∗∗^	5.91±0.80^∗∗∗^	7.22±1.83^∗∗∗^
FBG (mg/dl)								
4-week	97.00 ± 5.73	108.25 ± 4.05	115.33 ± 4.63^a^	125.50 ± 11.29	104.50 ± 3.95	98.25 ± 2.43^∗^	87.80±1.98^∗∗^	95.60 ± 1.81^∗^
8-week	94.80 ± 5.36	117.50 ± 4.29^aaa^	109.33 ± 1.86^a^	103.80 ± 3.20	107.25 ± 4.80	101.67 ± 2.73	95.67 ± 0.67^∗^	96.75 ± 2.02^∗^
Plasma glucose AUC (mg h/dl)								
4-week	287.13 ± 25.47	369.30 ± 30.77^aa^	355.75 ± 20.45^a^	355.30 ± 14.34	400.19 ± 16.76	298.88 ± 12.34	290.45 ± 19.06^∗^	311.70 ± 12.56
8-week	266.90 ± 10.34	346.29 ± 12.14^aa^	421.75 ± 29.80^aaa,bb^	350.50±13.04^∗∗^	360.25 ± 14.13^∗^	344.94±15.32^∗∗^	346.15±22.17^∗∗^	364.94 ± 21.94^∗^
ACE (units/mg.protein)	0.019 ± 0.001	0.028 ± 0.001^aaa^	0.028 ± 0.002^aaa^	0.020±0.002^∗∗^	0.020±0.001^∗∗^	0.022 ± 0.002^∗^	0.020±0.001^∗∗∗^	0.020±0.001^∗∗∗^

Data are presented as mean ± SEM (*n* = 6/group). ^a, aa, aaa^*p* value < 0.05, 0.01, and 0.001, respectively; compared to control rats, which received normal diet and vehicle, ^b, bb, bbb^*p* value <0.05, 0.01, and 0.001, respectively, compared to normal rats, which received HCHF diet and vehicle and ^∗^, ^∗∗^, ^∗∗∗^*p* value < 0.05, 0.01, and 0.001, respectively, compared to OVX rats, which received HCHF and vehicle.

**Table 4:** The effect of various doses of MME on serum estradiol level.

Treatment group	Estradiol (pg/ml)
ND + vehicle	25.39 ± 14.61
HCHF + vehicle	26.43 ± 9.46
OVX + HCHF + vehicle	5.00 ± 0.00^a,bb^
OVX + HCHF + isoflavone 15 mg/kg BW	8.15 ± 1.95
OVX + HCHF + L-carnitine 250 mg/kg BW	7.69 ± 1.81
OVX + HCHF + MME 10 mg/kg BW	5.00 ± 0.00
OVX + HCHF + MME 50 mg/kg BW	10.28 ± 5.28
OVX + HCHF + MME 250 mg/kg BW	11.24 ± 5.97

Data are presented as mean ± SEM (*n* = 6/group). ^a^*p* value<0.05, compared to control rats which received normal diet and vehicle; ^bb^*p* value < 0.01, compared to normal rats which received HCHF diet and vehicle. ND: normal diet; HCHF: high-carbohydrate high-fat diet; OVX+HCHF: ovariectomized rats which received high-carbohydrate high-fat diet; MME10, 50, and 250: the microencapsulated mulberry fruits extract at doses of 10, 50, and 250 mg/kg BW, respectively.

**Table 5:** Adiposity index of OVX rats with HCHF diet which received various treatments.

Treatment group	Adiposity index (%)
ND + vehicle	4.54 ± 0.66
HCHF + vehicle	11.25 ± 1.15^aaa^
OVX + HCHF + vehicle	12.80 ± 0.34^aaa^
OVX + HCHF + isoflavone 15 mg/kg BW	9.60 ± 0.63^∗^
OVX + HCHF + L-carnitine 250 mg/kg BW	7.29±0.89^∗∗∗^
OVX + HCHF + MME 10 mg/kg BW	10.43 ± 0.39
OVX + HCHF + MME 50 mg/kg BW	10.13 ± 0.68
OVX + HCHF + MME 250 mg/kg BW	9.25 ± 1.06^∗^

Data are presented as mean ± SEM (*n* = 6/group). ^aaa^*p* value < 0.001; compared to control rats which received normal diet and vehicle and ^∗^, ^∗∗∗^*p* value < 0.05 and 0.001, respectively, compared to OVX rats which received HCHF and vehicle.

**Table 6:** Oxidative stress markers in adipose tissue of OVX rats with HCHF diet which received various treatments.

Treatment group	MDA (ng/mg.protein)	SOD (units/mg.protein)	CAT (units/mg.protein)	GSH-Px (units/mg.protein)
ND + vehicle	0.52 ± 0.13	13.91 ± 2.21	375.89 ± 87.06	1.39 ± 0.36
HCHF + vehicle	1.10 ± 0.09^aaa^	7.95 ± 0.84^aaa^	269.48 ± 27.31^a^	1.01 ± 0.10^a^
OVX + HCHF + vehicle	1.64 ± 0.09^aaa,bb^	7.31 ± 1.28^aaa^	211.21 ± 3.89^aa^	0.79 ± 0.06^aa^
OVX + HCHF + isoflavone 15 mg/kg BW	1.26 ± 0.06^∗^	13.05±0.87^∗∗∗^	268.93 ± 24.81	1.22±0.13^∗∗^
OVX + HCHF + L-carnitine 250 mg/kg BW	0.76±0.17^∗∗∗^	10.96 ± 0.36^∗^	251.36 ± 9.15	1.01 ± 0.03
OVX + HCHF + MME 10 mg/kg BW	0.78±0.06^∗∗∗^	12.61±0.88^∗∗∗^	335.54±18.96^∗∗^	1.22 ± 0.06^∗^
OVX + HCHF + MME 50 mg/kg BW	1.00±0.14^∗∗∗^	9.96 ± 0.51^∗^	308.55 ± 16.36^∗^	1.20 ± 0.07^∗^
OVX + HCHF + MME 250 mg/kg BW	0.96±0.13^∗∗∗^	10.89±0.63^∗∗^	315.25 ± 19.00^∗^	1.19 ± 0.03^∗^

Data are presented as mean ± SEM (*n* = 6/group). ^a, aa, aaa^*p* value < 0.05, 0.01, and 0.001, respectively, compared to control rats which received normal diet and vehicle. ^bb^*p* value < 0.01, compared to normal rats which received HCHF diet and vehicle. ^∗^, ^∗∗^, ^∗∗∗^*p* value <0.05, 0.01, and 0.001, respectively, compared to OVX rats which received HCHF and vehicle.

## Data Availability

All the data (tables and figures) used to support the findings of this study are included within the article, and the details will be provided on request due to the registration of petty patent and the technology transfer agreement.
